# Combination Treatment of Resistant Acute Promyelocytic Leukemia Cells with Arsenic Trioxide and Anti-Apoptotic Gene Inhibitors

**DOI:** 10.3390/ph17111529

**Published:** 2024-11-14

**Authors:** Manuela Giansanti, Tiziana Ottone, Serena Travaglini, Maria Teresa Voso, Grazia Graziani, Isabella Faraoni

**Affiliations:** 1 Department of Systems Medicine, University of Rome Tor Vergata, 00133 Rome, Italy; manuela.giansanti@opbg.net; 2 Department of Biomedicine and Prevention, University of Rome Tor Vergata, 00133 Rome, Italyvoso@med.uniroma2.it (M.T.V.); 3 Unit of Neuro-Oncohematology, Santa Lucia Foundation-IRCCS, 00179 Rome, Italy

**Keywords:** APL, arsenic resistance, apoptosis, BCL2, venetoclax, BIRC3, SMAC mimetics, LCL161, xevinapant

## Abstract

Background: Arsenic trioxide (ATO) is an anticancer agent for treating acute promyelocytic leukemia (APL). However, 5–10% of patients fail to respond, developing relapsed/refractory disease. The aim of this study was to identify potential new therapeutic approaches for ATO-unresponsive APL by targeting the anti-apoptotic genes that contribute to drug resistance. Methods: RNA expression of dysregulated genes involved in the apoptotic pathway was analyzed by comparing ATO-resistant APL cell clones generated in our lab with the corresponding sensitive clones, at basal levels and after 48 h of treatment with ATO. Results: ATO-resistant APL cells showed upregulation of *APAF1*, *BCL2*, *BIRC3*, and *NOL3* genes, while *CD70* and *IL10* genes were downregulated, compared to ATO-sensitive cells. Treatment with ATO strongly increased the expression of the anti-apoptotic genes *BIRC3*, *NOL3*, and *BCL2A1* and significantly downregulated *BCL2* in ATO-sensitive clones. Although all these genes can be relevant to ATO-resistance, we selected BCL2 and BIRC3 as druggable targets. A direct correlation between *BCL2* expression and the sensitivity to the BCL2 inhibitor venetoclax was observed, indicating *BCL2* as predictive biomarker of the response. Moreover, the combination of venetoclax with ATO exerted synergistic cytotoxic effects, thus reverting the resistance to ATO. APL treatment with SMAC mimetics such as LCL161 and xevinapant (inhibitors of BIRC3) was not as effective as the BCL2 inhibitor as a monotherapy but exerted synergistic effects in combination with ATO in cells with low *BIRC* expression. Conclusions: This study demonstrates the therapeutic potential of venetoclax in combination with ATO in vitro and strongly encourages further investigation of relapsed/refractory APL with high *BCL2* expression.

## 1. Introduction

Acute promyelocytic leukemia (APL) is a subtype of acute myeloid leukemia (AML) with an aggressive clinical presentation, which is characterized by the reciprocal balanced translocation t(15;17) involving the promyelocytic leukemia (*PML*) and retinoic acid receptor alpha (*RARA*) genes. The PML-RARA oncoprotein blocks myeloid differentiation at the promyelocyte stage and induces aberrant self-renewal of APL cells with disruption of normal hematopoiesis [1]. Currently, all-trans retinoic acid (ATRA) plus arsenic trioxide (ATO), both acting as differentiating agents, represents the frontline treatment for non-high-risk APL [2,3]. The introduction of ATO in the management of APL patients has improved the overall survival and long-term quality of life in comparison with ATRA plus anthracycline-based chemotherapy [4,5]. Nonetheless, ATRA/ATO combination therapy can be associated with severe adverse effects, such as hyperleukocytosis and differentiation syndrome [2].

The main mechanism involved in ATO anti-leukemic effects relies on the degradation of PML-RARA molecular species, which results in apoptosis induction [6,7]. Moreover, ATO acts as an epigenetic regulator, modifying the DNA methylation pattern. Indeed, preclinical evidence reported that ATO inhibits DNA methyltransferases (DNMT1, DNMT3A, and DNMT3B) and restores the expression of methylation-silenced tumor suppressor genes [8].

Despite the high percentage of responses to the differentiating therapy, 5–10% of APL patients undergo disease relapse or fail to respond [2]. Multiple resistance mechanisms in relation to ATO have been reported [1,9]. For instance, a minor proportion of APL, also classified as AML with an APL-like morphology, does not have the typical *PML-RARA* fusion gene but chromosomal translocations involving different *RARA* gene partners (X-RARA chimeric transcripts such as PLZF-RARA [10], or NPM1-RARA fusion oncoproteins [11]) that are associated with a worse prognosis due to the lack of the ATO-binding region in the PML protein [1,11]. PML missense mutations within the B2 domain of the PML gene, such as the A216V/T mutation, may also result in ATO resistance, even though they are rarely detected at disease relapse [12,13]. Moreover, metabolic adaptation, dysregulation of redox signaling, altered expression of genes involved in survival pathways, autophagy, drug transport, cell cycle, and DNA damage repair machinery could also be implicated [1,9].

Apoptosis escape due to the upregulation of proteins implicated in the intrinsic (mitochondrial) pathway, including members of the B cell lymphoma 2 (BCL2) superfamily (e.g., BCL2, MCL1, BCL-xL/BCL2L1, BFL1/BCL2A1 anti-apoptotic proteins) and of the inhibitor of apoptosis (IAP) family (e.g., NAIP/BIRC1, cIAP1/BIRC2, cIAP2/BIRC3, XIAP/BIRC4, and survivin/BIRC5), may contribute to the development of drug resistance in many cancer types, including AML [14,15,16,17]. Consequently, targeting deregulated apoptotic pathways represents a promising strategy for treating hematological malignancies that fail to respond to apoptosis-inducing agents. Presently, the highly selective BCL2 inhibitor venetoclax (ABT-199) is indicated for the treatment of adult patients with chronic lymphocytic leukemia (CLL) and small lymphocytic lymphoma (SLL) [18] and, in combination with decitabine or azacitidine or low-dose cytarabine, for AML patients older than 75 years or unfit for intensive induction chemotherapy. IAPs can be targeted by small molecules that mimic an endogenous inhibitor called the second mitochondria-derived activator of caspase (SMAC/DIABLO) [19]. Indeed, SMAC mimetics (or IAP antagonist drugs) are under clinical investigation for hematological malignancies and solid tumors [https://clinicaltrials.gov/ (accessed on 9 September 2024)].

Characterization of gene expression involved in apoptosis in the context of ATO-resistant APL might lead to identifying novel targeted approaches for this clinical condition. In the present study, we have adopted an in vitro model of ATO-resistant APL cells [7], without ATO-binding domain mutations, to explore potential alternative drugs for relapsed/refractory patients. The expression pattern of genes involved in the apoptotic pathways was determined to identify altered genes related to ATO resistance (i.e., *APAF1*, *BCL2*, *BCL2A1*, *BIRC3*, *CD70*, *IL10*, *NOL3*). ATO-resistant cells overexpressing *BCL2* responded to the BCL2 inhibitor venetoclax. On the contrary, hitting IAPs with the SMAC mimetics LCL161 and xevinapant was effective in some APL clones, regardless of whether they were sensitive or resistant to ATO.

## 2. Results

### 2.1. Comparative Expression Pattern of Apoptotic Genes in ATO-Sensitive and ATO-Resistant APL Cells

Clones from the human promyelocytic leukemia cell line NB4 were generated in our laboratory by limiting dilution (CL1, CL2, CL3, and CL4) and then individually exposed to increasing concentrations of ATO, thus obtaining corresponding ATO-resistant clones (CL1-R, CL2-R, CL3-R and CL4-R), as previously reported [7]. Interestingly, reduced apoptosis activation was observed in the resistant clones after treatment with ATO [7].

**Figure 1:**
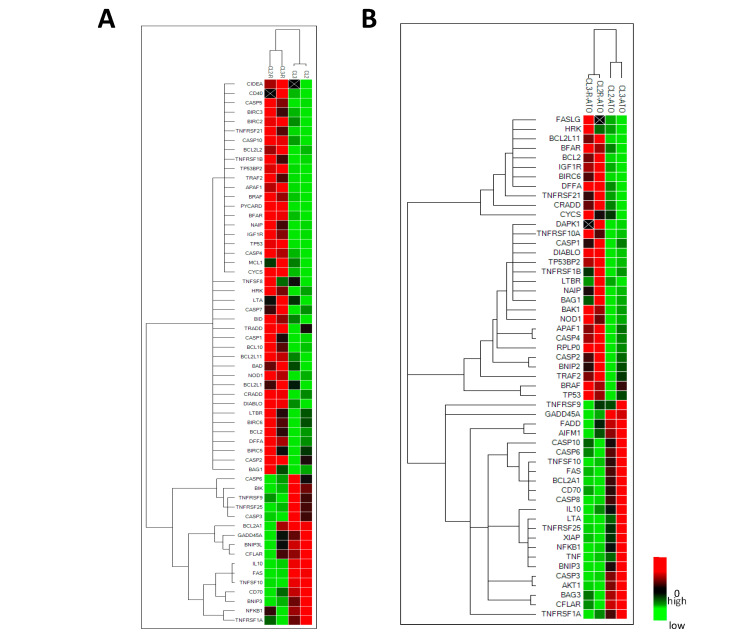

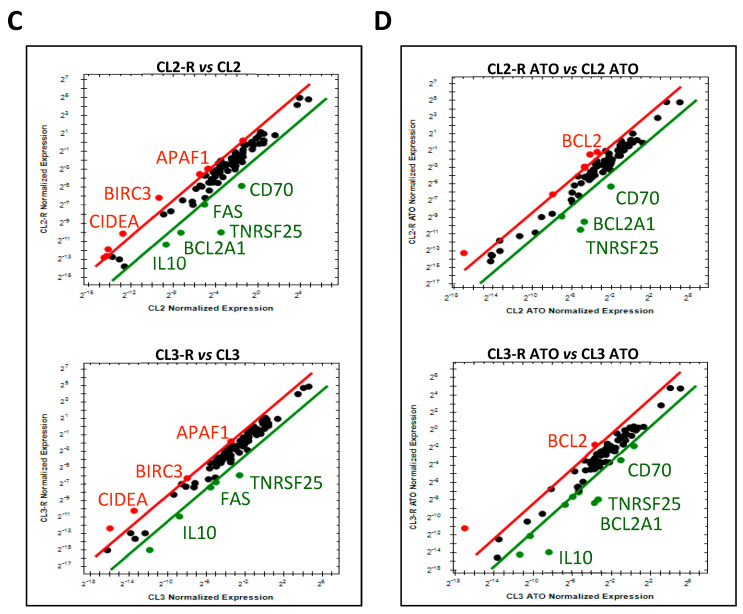
Differential apoptotic gene expression in ATO-resistant and -sensitive APL cell lines. Expression of 82 apoptotic genes in CL2-R and CL3-R sensitive clones was compared with that observed in CL2 and CL3 parental clones, respectively. The clustergrams reveal the different pattern of RNA expression found in untreated growing cells (**A**) and in cells treated with ATO for 48 h (**B**). In the graphs, ≥3–fold dysregulated genes are shown in untreated growing cells (**C**) and ATO-treated cells (**D**).

**Figure A1:**
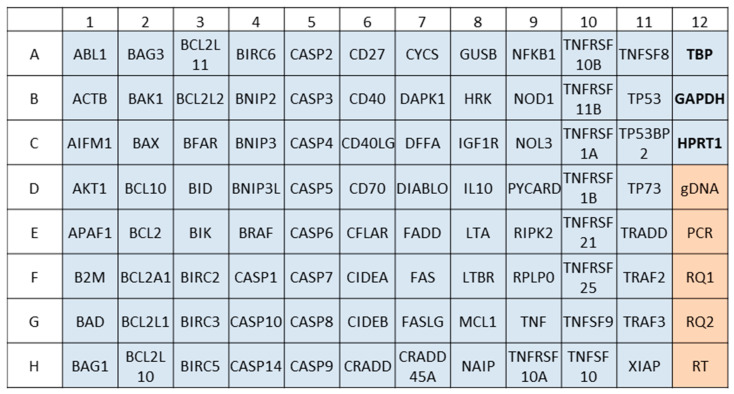
Apoptotic genes list. A custom apoptosis PrimePCR Pathway Plate was used to analyze the 82 apoptosis-related genes (the blue squares). Three housekeeping genes were used to normalize the data and are represented in bold (TATA-box-binding protein, TBP; glyceraldehyde-3-phosphate dehydrogenase, GAPDH; hypoxanthine phosphoribosyltransferase 1, HPRT1). The predesigned plate also included PCR controls: gDNA as genomic DNA contamination control, PCR as a positive qPCR assay control, RQ1 and RQ2 as RNA integrity quality controls, RT as reverse transcriptase performance assay control.

Herein, to identify critical apoptotic genes involved in ATO resistance that could potentially be pharmacologically targeted, we used a real-time PCR array gene expression profile kit for detecting 82 apoptotic genes (Figure A1 in Appendix A). Comparative analysis of the basal levels of gene expression was carried out on two pairs of NB4 clones (CL2/CL2-R and CL3/CL3-R) without treatment (Figure 1A) and after 48 h of exposure to 1 μM ATO, a commonly accepted concentration tested in in vitro studies (Figure 1B). In the heatmaps, genes that showed a substantial difference in the expression level between the ATO-sensitive and ATO-resistant clones were clustered. Finally, we selected the ≥3-fold deregulated genes at basal levels (*APAF1*, *BCL2A1*, *BIRC3*, *CD70*, *CIDEA*, *FAS*, *IL10*, *TNRSF25*, Figure 1C) or after ATO treatment (*BCL2*, *BCL2A1*, *CD70*, *IL10*, *TNRSF25*; Figure 1D) and these genes were further investigated in sensitive and resistant clones by real-time PCR (RT-PCR) by using previously validated primers (Table A1). In regard to the *NOL3* gene, the software did not analyze it (in fact, it does not appear in the figure) since it was undetectable in the sensitive clones. However, *NOL3* was detected in the ATO-resistant cells in basal conditions and upregulated after pharmacological treatment. Therefore, we considered this difference of interest and included the *NOL3* gene in the subsequent analysis.

**Figure 2:**
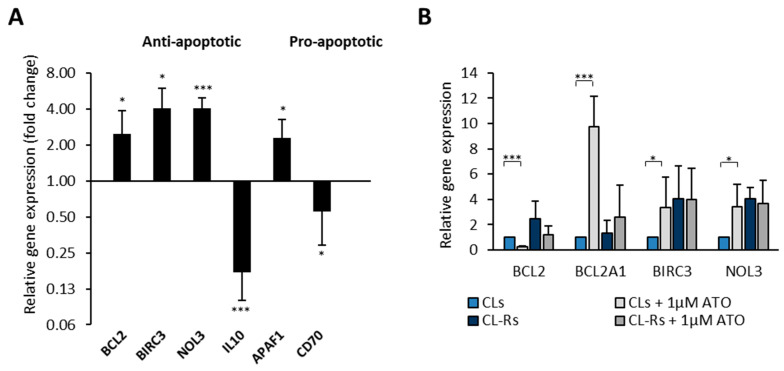
Modulation of anti-apoptotic and pro-apoptotic gene expression in ATO-resistant clones. The RT-PCR analysis of the ATO-resistant clones was compared with that of their ATO-sensitive counterparts using the primers reported in Table A1. The mean relative expression was obtained by normalizing with GAPDH and calibrating each resistant clone against its parental clone. CLs: mean values for RNA expression in the four ATO-sensitive clones; CL-Rs: mean values for RNA expression in the four ATO-resistant clones. (**A**) The histogram shows the six genes significantly modulated in basal conditions among the previous nine. (**B**) The histogram shows the four genes significantly modulated after 48 h of ATO treatment. Statistical analysis was performed using the unpaired Student’s *t*-test: * *p* < 0.05, *** *p* < 0.001.

The following analysis confirmed the upregulation of the anti-apoptotic genes *BCL2*, *BIRC3*, and *NOL3* in the ATO-resistant cells and the downregulation of *IL-10* (>2-fold). Among the pro-apoptotic genes, *APAF1* was also upregulated in these cells and *CD70* downregulated (>2-fold) (Figure 2A). Regarding the modulation of gene expression after ATO pharmacological treatment, BCL2 expression was reduced in both the sensitive and resistant cells compared to their untreated counterparts. However, overall, the *BCL2* level in the unresponsive clones remained higher than in the sensitive ones. The expression of the *BCL2A1*, *BIRC3*, and *NOL3* anti-apoptotic genes was markedly increased after treatment with ATO in only the ATO-sensitive clones (9.7-, 3.4-, and 3.4-fold, respectively). In contrast, it was unchanged in the resistant ones (Figure 2B). With the aim of finding potential new therapeutic approaches for treating APL that is unresponsive to ATO, we explored the overexpression of *BCL2* and *BIRC3* as pharmacological targets. In fact, BCL2 and BIRC3 may promote the survival/growth of ATO-resistant cells and selective inhibitors of BCL2 (e.g., venetoclax) or IAP proteins (SMAC mimetic) are already used in clinics.

### 2.2. High Expression of BCL2 in APL Cells Directly Correlates with Venetoclax Sensitivity

**Figure 3:**
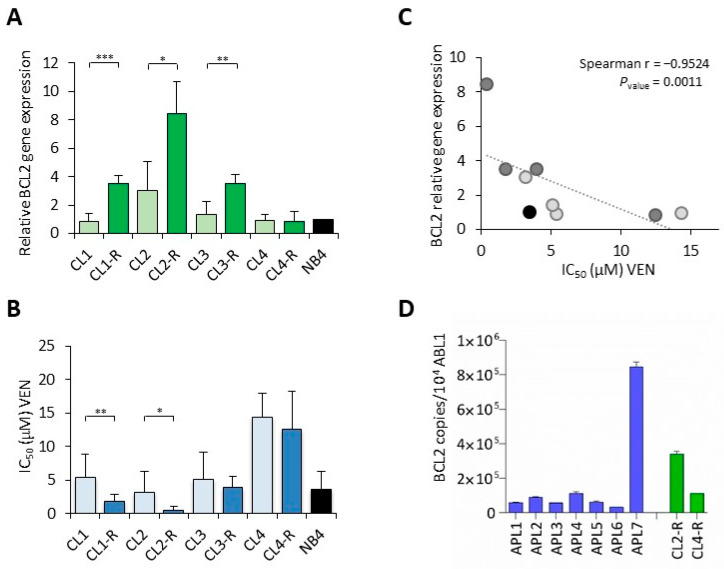
*BCL2* expression correlates with venetoclax sensitivity. (**A**) RNA expression of *BCL2* was measured by RT–PCR analysis of the total RNA extracted from cultured NB4 cell clones, using three distinct RNA preparations extracted from cells collected at different times. Relative expression was obtained by normalizing with GAPDH and calibrating with the NB4 bulk cell line. Data are presented as the mean of triplicate experimental analysis ± SD. Statistical analysis was performed using the unpaired Student’s *t*-test: * *p* < 0.05, ** *p* < 0.01, *** *p* < 0.001. Differences between sensitive clones were not statistically significant. (**B**) ATO-sensitive cell clones (light blue), ATO-resistant cell clones (bright blue), and the NB4 cell line (black) were treated with increasing concentrations of venetoclax (0.01, 0.1, 1, 10 μM) and analyzed by MTS test. The surviving fractions were compared to the untreated control at 48 h after drug exposure. Values are the mean values ± SD of at least three independent experiments. Statistical analysis was performed using the unpaired Student’s *t*-test. (**C**) Relationship between RNA *BCL2* expression and venetoclax (VEN) IC_50_ values in NB4 clones and the NB4 bulk cell line. ATO-sensitive cell clones (light gray), ATO-resistant cell clones (dark gray), and the NB4 cell line (black). The non-parametric Spearman’s correlation coefficients and the significance level are indicated. (**D**) Levels of *BCL2* transcripts evaluated by qRT–PCR as number of copies and normalized to ABL1 copies in the mononuclear cells of APL patients at diagnosis (n = 7) and in CL2-R and CL4-R ATO-resistant clones.

Exponentially growing cultured cells from all the previously generated NB4 ATO-sensitive and resistant clones were harvested to analyze the expression of *BCL2* mRNA (Figure 3). At the basal level, three out of four ATO-resistant clones showed a significant upregulation of *BCL2* compared to the parental ones, suggesting that the increased expression of this anti-apoptotic gene is a frequent finding (Figure 3A). To investigate the potential activity of the BCL2 inhibitor venetoclax in APL cells, ATO-sensitive and ATO-resistant clones, as well as the NB4 bulk cell line, were exposed to increasing concentrations of the drug. Cell growth was first analyzed by the MTS assay after 48 h of a single exposure to venetoclax and the concentrations capable of inhibiting 50% of the cell growth (IC_50_) values are reported in Figure 3B. The drug concentrations tested in the experiments always included the plasma C_max_ reported in cancer patients during phase 1 clinical trials (steady state C_max_ range = 1.8–2.5 μM) [20]. All the ATO-resistant clones with higher *BCL2* expression were more sensitive to venetoclax with lower IC_50_ values (range 0.3–3.3 µM) compared to the corresponding parental clones (range 3.2–6.4 µM). On the contrary, no increase in BCL2 expression and no difference in venetoclax sensitivity were observed between the CL4 and CL4-R clones. Of interest, the basal levels of *BCL2* expression were variable among the clones and directly correlated with the cell sensitivity to venetoclax (*p* = 0.0011, Spearman’s Rho correlation: −0.9524) (Figure 3C). The ATO-resistant clones overexpressing *BCL2* showed IC_50_ values that were in the range of plasma levels detected in clinical studies [20,21]. On the contrary, the CL4 clone and its CL4-R counterpart with very low basal *BCL2* levels did not respond to clinically relevant concentrations of venetoclax. These results indicated that the sensitivity to venetoclax of the ATO-resistant NB4 APL cells depends on the BCL2 expression levels.

Our experimental model highlights that the cellular response following repeated drug treatments can be heterogeneous in vitro. This model may partially reproduce the heterogeneity that can be found in primary APLs. To verify these findings in the clinic, we analyzed the mRNA expression of *BCL2* in seven APL primary mononuclear cell cultures collected from leukemia patients at disease presentation by quantitative RT-PCR (qRT-PCR). A high expression of *BCL2* was observed in only one case (Figure 3D), confirming that the expression of BCL2 can be highly different from one patient to another. Based on these observations, we speculated that differences in the expression of *BCL2* might account, at least in some cases, for resistance to ATO and sensitivity to venetoclax.

### 2.3. Synergistic Cytotoxic Effects of ATO with Venetoclax

**Figure 4:**
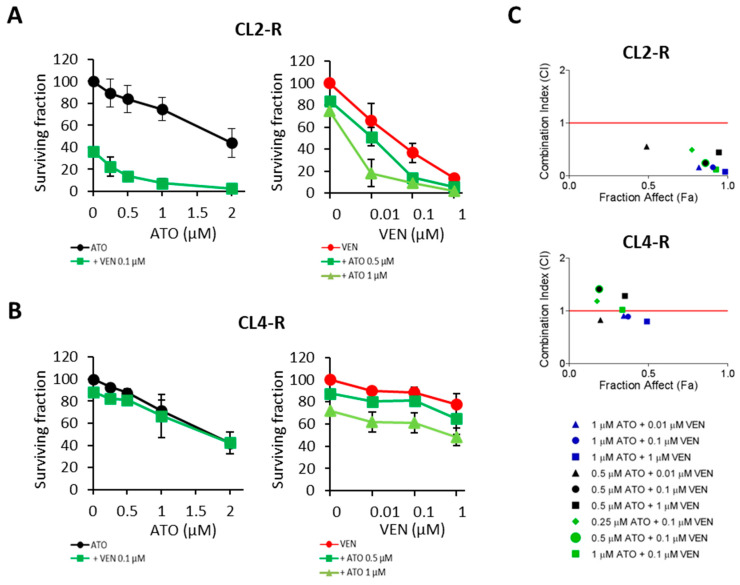
Combined treatment of ATO and venetoclax induces synergistic growth inhibitory effects in cells with high *BCL2* expression. ATO-resistant CL2-R (**A**) and CL4-R (**B**) clones were treated with the indicated concentrations of arsenic trioxide (ATO) with or without a fixed concentration (0.1 μM) of venetoclax (VEN) (left panels) or with the indicated concentrations of VEN and with or without a fixed concentration (0.5 and 1 μM) of ATO (right panels). After three days, proliferation was evaluated by cell count in triplicate. Data are represented as surviving fractions. Values are the mean ± SD of three independent experiments. (**C**) ATO plus VEN combined effects were analyzed in CL2-R (upper panel) and in CL4-R (bottom panel) clones using CompuSyn software version 1.0. Each Fa-CI plot (or Chou–Talalay plot) indicates the CI as a function of the fraction affected (Fa). CI < 1, synergistic (values below the dotted line); CI = 1, additive; CI > 1, antagonist.

As shown in Figure 2B, treatment with ATO induced downregulation of *BCL2* in both sensitive and resistant cells. However, resistant cells did not reach the low *BCL2* expression found in sensitive cells. Since the persistence of high BCL2 may prevent the occurrence of apoptosis in ATO-resistant cells, we assumed that a reduction of BCL2 activity induced by venetoclax could facilitate the cytotoxic effects of ATO. To assess the efficacy of venetoclax in combination with ATO, CL2-R (very high *BCL2*) and CL4-R (very low BCL2) cells were exposed to increasing concentrations of ATO (0.25, 0.5, 1, and 2 µM) plus a fixed dose of venetoclax (0.1 µM) (Figure 4A,B, left panels), or to increasing concentrations of venetoclax (0.01, 0.1, and 1 µM) plus a fixed dose of ATO (0.5 or 1 µM) (Figure 4A,B, right panels). Analysis of the cell proliferation by cell count, performed after 72 h of treatment, confirmed the trend observed with the MTS technique: the response to ATO was comparable between the two clones, whereas the venetoclax IC_50_ values were 0.04 and 3.9 µM for CL2-R and CL-4, respectively. As assessed by the CompuSyn method [22], synergistic effects were only found in CL2-R cells (Fa-CI plots of Figure 4C) at all the tested drug combinations, with combination index (CI) values largely below 1, but not in the CL4-R clone in which *BCL2* was minimally expressed.

### 2.4. SMAC Mimetics Induce Growth Inhibitory Effects in CL1-R

IAP genes have been found to be deregulated in several tumors, including AML [14]. Among these, XIAP, BIRC2 (cIAP1), and BIRC3 (cIAP2) are known to play a direct role in the control of apoptosis by inhibiting caspase activation [23]. SMAC/DIABLO is a mitochondrial-derived pro-apoptotic protein released into the cytosol upon apoptotic stimulation. It promotes apoptosis by binding and blocking the activity of IAPs (BIRC2/3 and XIAP) and promoting their degradation. Therefore, SMAC mimetics can facilitate the activation of caspase 3, 7, and 9 by binding and neutralizing IAP proteins.

The RNA analysis showed higher *BIRC3* expression in ATO-resistant cells than in parental cells when examined by both PCR array and RT-PCR (Figure 1 and Figure 2A). However, significant upregulation following ATO treatment was only observed in ATO-sensitive clones (Figure 1 and Figure 2B).

**Figure 5:**
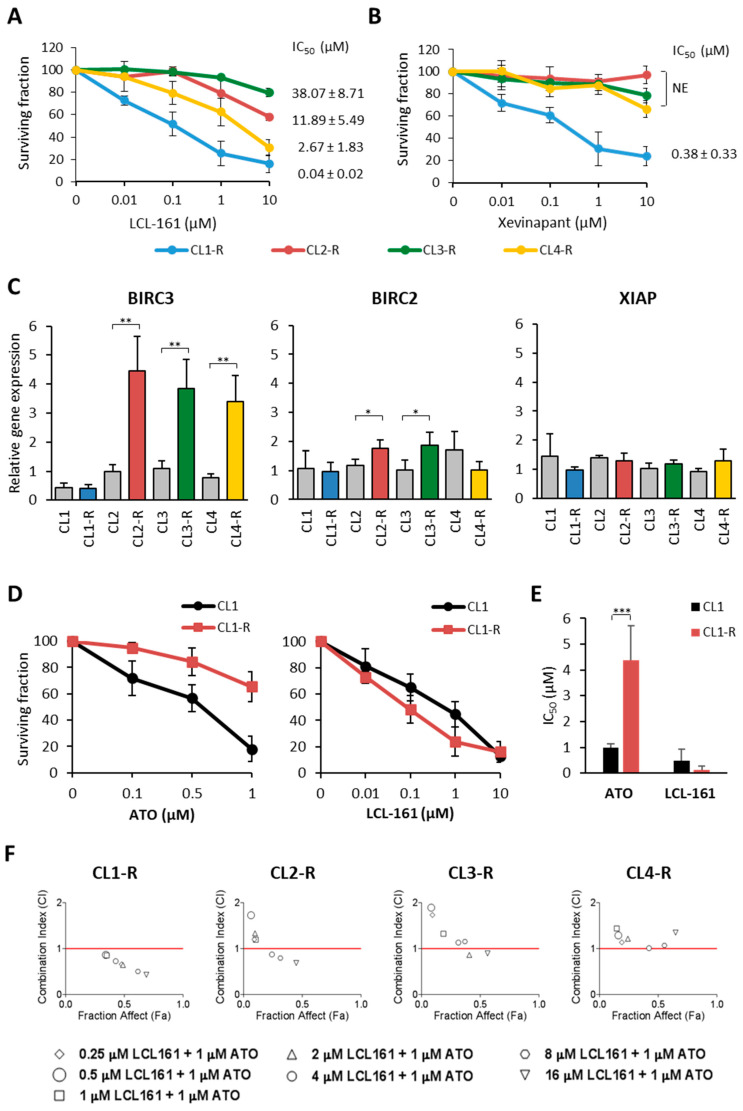
SMAC mimetics’ growth inhibitory effect is independent of ATO resistance and IAPs RNA expression. ATO-resistant clones were treated with the indicated concentrations of LCL161 (**A**) or xevinapant (**B**). After three days, the proliferation was evaluated by cell count in triplicate. Data are represented as surviving fractions and are the mean ± SD of three independent experiments. The IC_50_ for each cell clone is indicated on the right of each graph. NE = not evaluable. (**C**) RNA expression of *BIRC3*, *BIRC2*, and *XIAP* was measured by RT-PCR using the total RNA extracted from cultured cell clones. Relative expression was obtained by normalizing to GAPDH and calibrating to the NB4 bulk cell line. Data are presented as the mean ± SD of triplicate RNA extractions. (**D**) CL1 and CL1-R cells were treated for three days with the indicated concentrations of ATO (left panel) or LCL-161 (right panel). Data are represented as surviving fractions and are the mean ± SD of three independent experiments evaluated by cell count. (**E**) IC_50_ mean values are shown. (**F**) ATO-resistant clones were treated with 1 µM of ATO plus graded concentrations of LCL161 (0.25–16 µM). The combined effects were evaluated in the four ATO-resistant clones after 48 h of drug treatment by MTS test and analyzed using CompuSyn software. Each Fa-CI plot (or Chou–Talalay plot) indicates the CI as a function of the fraction affected (Fa). CI < 1, synergistic (values below the dotted line); CI = 1, additive; CI > 1, antagonist. Statistical analysis was performed using the unpaired Student’s *t*-test: * *p* < 0.05, ** *p* < 0.01, *** *p* < 0.001.

The high expression of anti-apoptotic BIRC3 might induce resistance to ATO that could potentially be blocked by SMAC mimetics [24]. NB4 ATO-resistant clones were then treated with two different SMAC mimetics: LCL161, an inhibitor of mainly BIRC2/3, and xevinapant, an inhibitor of both BIRC2/3 and XIAP. Cells were exposed to increasing concentrations (0.01–10 µM) of LCL161 (Figure 5A) and xevinapant (Figure 5B) and proliferation was analyzed by cell count after 72 h. The CL1-R clone was the most sensitive to both SMAC mimetic drugs, whereas the other clones showed a low sensitivity (CL4-R) or were totally unresponsive (CL2-R and CL3-R). To verify whether these substantial differences in the sensitivity to the SMAC inhibitors had to be attributed to different expression levels of IAPs, exponentially growing untreated cells were harvested and analyzed for the expression of *BIRC3, BIRC2*, and *XIAP* by RT-PCR (Figure 5C). Among the ATO-resistant clones, *BIRC3* expression remained low in CL1-R and was comparable to its parental counterpart, *BIRC2* was slightly overexpressed in CL2-R and CL3-R, whereas *XIAP* showed no significant difference among the ATO-sensitive and ATO-resistant clones (Figure 5C). These data were in line with the results obtained by the PCR array profiling. No statistically significant correlation was observed between the IAPs mRNA expression and the SMAC inhibitors IC_50_ values. Then, we compared the sensitivity of the CL1 and CL1-R cell clones to ATO and LCL161 drugs. After 72 h of treatment, CL1 was highly responsive to both drugs, whereas CL1-R was highly sensitive to only LCL161 (Figure 5D,E). These data suggest an intrinsic susceptibility of these cell clones to the SMAC inhibitor, independently of ATO resistance. To discern whether the three clones showing upregulation of *BIRC3* acquired resistance to ATO as a consequence of an increase in BIRC3 or other IAPs expression, we hypothesized that the sensitivity to ATO could be restored by inhibiting IAPs. To this end, we tested the cell proliferation of ATO-resistant clones after treatment with graded concentrations of LCL161 (0.25 to 16 µM) in combination with a fixed ATO dose (1 µM) at 48 h by MTS. Synergic effects were observed in CL1-R with all the tested combinations (Figure 5F). On the contrary, the other clones did not benefit from the blockade of the anti-apoptotic proteins’ IAPs. Thus, upregulation of *BIRC3* in ATO-resistant clones did not appear to be directly responsible for the acquisition of an ATO-resistant phenotype.

## 3. Discussion

The identification of additional druggable targets is of crucial importance to establishing adequate therapeutic options to be offered to cancer patients at disease relapse. In this study, we analyzed the expression pattern of genes involved in apoptosis on APL cell clones resistant to ATO to identify proteins potentially implicated in the resistance phenotype that could be inhibited by pharmacologically active small molecules for ATO-relapsed/refractory APL.

Our study first analyzed the RNA expression in basal conditions and after exposure to ATO of sensitive and resistant APL cells. Since in leukemia cells ATO activates the apoptotic pathway by oxidative stress, DNA damage, and lowering the mitochondrial membrane potential, we have focused our search on genes involved in these processes [25]. We found significant differences in cells unresponsive to ATO compared to the parental clone, with the *BCL2*, *BIRC3*, and *NOL3* anti-apoptotic genes being overexpressed and *IL-10* underexpressed in ATO-resistant cells; among the pro-apoptotic genes, *APAF1* was overexpressed and *CD70* downregulated. Based on the considerations discussed below, among these genes, we have chosen *BCL2* and *BIRC3* as the most pharmacologically targetable.

In ATO-resistant clones, we observed a reduced expression of *IL-10*, a pleiotropic anti-inflammatory cytokine that acts on many immune cell types by repressing the expression in macrophages of molecules that are often activators of cell death, such as TNF-alpha, IL-6, and IL-1-beta [26]. Through stimulation of its receptor, IL-10 was also shown to directly promote the survival of progenitor myeloid cells by stimulating phosphorylation of the insulin receptor substrate-2 (IRS2) and PI3-kinase/Akt pathways [27]. Overall, due to the complex crosstalk between the immune component of the tumor microenvironment and leukemia cells, the outcome of IL-10 pharmacological modulation is difficult to predict.

In ATO-resistant clones, the basal level of *CD70* was downregulated compared to the ATO-sensitive ones. The immune checkpoint molecule CD70 and its receptor CD27 belong to the tumor necrosis factor (TNF) superfamily and promote T-lymphocyte expansion and differentiation. Aberrant activation of the CD70–CD27 axis within the tumor and its microenvironment accelerates cell proliferation and immunosuppression and is involved in AML progression [28,29]. The discovery of the tumor-promoting and immunosuppressive effects of the CD70–CD27 axis has revealed novel targets and stimulated new preclinical therapeutic strategies [29,30,31]. However, the function of CD70 concerning apoptosis in cancer is complex and varies depending on the tumor microenvironment.

The *NOL3* gene (previously known as the apoptosis repressor with caspase recruitment domain (ARC)) was overexpressed in ATO-resistant cells in basal conditions and upregulated after ATO treatment. NOL3 suppresses both extrinsic and intrinsic apoptotic pathways and is associated with abnormal cell growth, invasiveness, and chemoresistance [32]. In AML patients, NOL3 is a prognostic factor that predicts adverse outcomes [33,34]. Its deletion in a transgenic mouse model of breast cancer reduced the primary tumor burden and limited cell invasion. Moreover, NOL3 overexpression induced chemoresistance to doxorubicin in vitro and in vivo [35]. Thus, NOL3 might provide a potential target for future anticancer therapies, but to date, no inhibitors of this protein have been marketed.

The decrease of *APAF1* expression following DNA methylation or deactivation of the protein is common in different cancer types [36]. In our APL-resistant model, an increase in the expression of the essential apoptosis regulator *APAF1* was observed, which could be explained by an alteration in the DNA methylation pattern induced by ATO. However, based on the pro-apoptotic effects of this protein, its overexpression in resistant cells is unlikely to play a key role in the ATO resistance mechanism.

Of particular interest was the increase we observed in *BCL2* expression in ATO-resistant APL cells. This molecular alteration might represent a potential pharmacological target for APL since a drug inhibiting BCL2 is already approved for AML treatment (i.e., the small molecule, BH3 mimetic drug venetoclax) [3,37]. ATO-induced cell death has been associated with BCL2 downregulation [25], a finding also confirmed by our results after the treatment of ATO-sensitive APL clones (Figure 2B). Indeed, the higher expression of BCL2 in ATO-resistant cells might explain their lower ability to undergo apoptosis. In AML, an inverse correlation between high BCL2 expression and the response to cytosine arabinoside and anthracyclines chemotherapeutic regimes was also reported [38,39]. However, both CL4 and CL4-R APL cells express a low *BCL2* level, although they show substantial differences in sensitivity to ATO (IC_50_ = 0.88 vs. 4.53 µM; [7]), suggesting that other mechanisms can induce resistance to ATO.

In our cellular model, three out of four ATO-resistant NB4 clones showed higher *BCL2* expression compared to the parental ones (Figure 3). Interestingly, following venetoclax treatment, we found an inverse correlation between the venetoclax IC_50_ values and *BCL2* RNA expression (*p* = 0.0011). Thus, based on our results and the drug’s mechanism of action, a high expression of BCL2 seems to be common in ATO APL-resistant cells but also required, in this leukemia type, for sensitivity to venetoclax, suggesting that BCL2 expression might be regarded as a biomarker of the response. The preliminary analysis of the *BCL2* mRNA levels in samples from APL patients at onset highlights the elevated expression of *BCL2* in one sample out of seven, indicating a certain variability among patients. The heterogeneity of anti-apoptotic dependencies across patients [15,40], as a consequence of selective pressure leading to the upregulation of pro-survival gene products, via genetic and non-genetic mechanisms, has already been shown in other, more common hematological malignancies (AML, DLBCL, etc.) [41,42].

In AML, the biological and clinical efficacy of venetoclax as a single agent has produced unsatisfactory results, leading to the approval of venetoclax in combination with DNA methyltransferase inhibitors or low-dose cytarabine [43,44,45]. Furthermore, about 69% of patients do not respond to frontline venetoclax plus hypomethylating agents or eventually relapse [46]. Therefore, there is an urgent need to identify molecular markers to predict the response/resistance to venetoclax and develop drug combination strategies. We designed primers and probe sequences for qRT-PCR analysis of RNA expression (Figure 3D, Table A2) as this is an acceptable and practical technique for evaluating BCL2 as a response biomarker in primary samples. Interestingly, venetoclax plus ATO in our APL cell models exerted synergistic cytotoxic effects in the ATO-resistant clone showing the highest *BCL2* basal levels (CL2-R). The combined treatment was not able to revert resistance to ATO when the *BCL2* expression was low (CL4-R), again suggesting BCL2 to be a potential biomarker of drug sensitivity. To the best of our knowledge, some clinical cases of APL patients carrying X-RARA translocations [47,48,49,50] or being refractory to standard chemotherapy [51] were successfully treated with venetoclax, either as a single agent or in association with cytarabine and anthracycline. In pediatric APL, substituting traditional cytoreduction therapy with venetoclax led to encouraging results in terms of efficacy as well as safety [52]. In future studies, it would be interesting to investigate *BCL2* expression on high-risk primary samples resistant to drug treatment with an ATRA/ATO regimen to delve into the dependencies of clinical APL on the pro-survival BCL2 gene across patients and provide evidence of the translational meaning of our in vitro results

Herein, we found that *BIRC3* was expressed at high levels in ATO-resistant clones, while other IAP-encoding genes (*BIRC2*, *XIAP*) did not appear to be modulated. IAPs regulate apoptosis by inhibiting caspases or regulating different transcriptional pathways by their ubiquitin E3 ligase activity, including canonical and non-canonical NF-kB pathways [53,54]. BIRC3 is an apoptosis inhibitor containing three consecutive BIR domains, zinc-binding folds that mediate protein–protein interaction. Due to its numerous activities, the role of BIRC3 in cancer is highly context-dependent. Indeed, deletion (78%) or mutation (8%) of BIRC3 is common among 11q22.3-q23.1 (11q-) CLL patients and is associated with less favorable outcomes [55], possibly because inactivation of the *BIRC3* gene leads to the constitutive activation of the non-canonical NF-kB pathway [56]. On the other hand, chromosomal amplification or overexpression of *BIRC3* was observed in various tumors, including glioblastoma and renal cell, gastric or small- and non-small-cell lung carcinomas [53,57]. In AML, high transcript levels were associated with poor overall survival [58]. The BIRC3 protein and the other type II BIR IAPs are regulated and inhibited by SMAC/DIABLO, a mitochondrial protein released into the cytosol when cells undergo apoptosis, which promotes cytochrome c-dependent caspase activation by eliminating IAP inhibition. In the present study, we used LCL161 and xevinapant (formerly AT406 and Debio1143), two SMAC mimetics under clinical investigation for leukemia and other tumor types [https://clinicaltrials.gov/ (accessed 9 September 2024)]. Among the resistant APL clones, CL1-R was highly sensitive to both drugs, although the *BIRC3* RNA expression was low. The lack of a direct correlation between the IAPs levels and the response to SMAC mimetics suggests that more complex mechanisms are involved in the antitumor activity of these anticancer agents, rendering difficult the identification of biomarkers to predict the tumor response.

Altogether, this study emphasizes the importance of identifying APL’s dependency on anti-apoptotic proteins for non-responder patients. This is particularly challenging considering the high heterogeneity of anti-apoptotic gene expression across cancer patients [15,40]. Nevertheless, our study suggests a strong potential of venetoclax in combination with ATO for relapsed/refractory APL that is unresponsive to conventional therapy following BCL2 expression evaluation.

## 4. Materials and Methods

### 4.1. Cell Lines and Cell Culture Conditions

The eight NB4 cell clones used in this work were generated from the APL cell line NB4 (American Type Culture Collection, ATCC) in our laboratory, as previously described [7]. Briefly, four different clones (CL1, CL2, CL3, and CL4) were produced by limiting dilution from the NB4 cell line during the early passages. To generate ATO-resistant clones, cells were independently exposed to increasing concentrations of ATO (0.1–1 μM) within a timeframe of one year. After about one year, the corresponding ATO-resistant clones were obtained and named CL1-R, CL2-R, CL3-R and CL4-R. Several cell aliquots were frozen during early passages. The ATO IC_50_s in the drug-sensitive clones, after three days of treatment, were in the range of 0.5–0.9 µM. At the same time, the IC_50_ values for the ATO-resistant clones increased to 2.6 μM, 4.5 μM, 2.7 μM and 4.5 μM for CL1-R, CL2-R, CL3-R and CL4-R, respectively.

As reported in a previous article, the ATO-sensitive and ATO-resistant clones maintained the expression of the fusion *PML/RARA* gene without mutations in the ATO-binding B2 domain. No correlation was found between ATO sensitivity and the cell proliferation rate.

All the clones were cultured in RPMI-1640 medium (Sigma-Aldrich, Merk, Darmstadt, Germany) supplemented with 2 mM L-glutamine (EuroClone, Milan, Italy), 1% penicillin/streptomycin (EuroClone) and 20% fetal bovine serum (Sigma-Aldrich) at 37 °C in a humidified CO_2_ incubator. During the in vitro culture, the ATO-resistant clones were maintained with 1 µM of ATO.

The RNA samples from APL mononuclear cells were obtained from bone marrow aspirates at the time of diagnosis and were available in our biobank at the Institute of Hematology of the University of Rome Tor Vergata [59]. ATRA chemotherapy was used as first-line treatment in all the patients.

### 4.2. Drug Treatment and Survival Assay

The aliquots of stock solution of ATO (As_2_O_3_, Sigma-Aldrich) were prepared by dissolving the drug in 1 N NaOH and then diluting it in PBS (EuroClone) to a final concentration of 2 mM. For each experiment, an aliquot was thawed, diluted with RPMI medium at 200 µM, and stored for a further month in a refrigerator at 4 °C.

The stock solutions of venetoclax, LCL161, and xevinapant (Selleck Chemicals, Houston, TX, USA) were prepared by dissolving the powder of each compound in DMSO (Sigma-Aldrich) at 20 mM. The drug aliquots were stored at −80 °C. For each experiment, a new aliquot was thawed and used. In all the experiments, the DMSO final concentration in the culture medium was always lower than 0.1% (*v*/*v*). For the cell treatment, the drugs were added at the beginning of each experiment and left in the culture medium until cell harvesting.

For the survival assays, the cells were analyzed by the MTS metabolic test (CellTiter 96 Aqueous One Solution Cell Proliferation Assay, Promega, Madison, WI, USA), according to the manufacturer’s instructions, or by the cell count viability test with trypan blue dye. Compared to the untreated control, the drug concentration capable of inhibiting 50% of cell growth (IC_50_) was extrapolated from the dose–response curves using linear regression (GraphPad Prism 5 software). The dose–effect curves of the drug co-treatments were analyzed by the Chou and Talalay method with the CompuSyn software (ComboSyn Inc. Paramus, NJ, USA) using the mean of three experiments. The combination index (CI) indicates a quantitative measure of the drug combination effects in terms of the synergistic (CI < 1), additive (CI = 1), or antagonistic effect (CI > 1).

### 4.3. PCR Array Profiling and RNA Expression Analysis

The total RNA was isolated with 1 mL of TRIzol reagent (Invitrogen, Thermo Fisher Scientific, Whaltham, MA, USA) and purified by Direct-zol RNA miniprep (Zymo Research, Irvine, CA, USA). For the PCR array profiling, 1 µg of RNA was reverse transcribed in 20 µL RT reaction with iScript reaction mix (Bio-Rad, Milan, Italy) containing dNTPs, oligo(dT), random primers, MgCl_2_, enhancers, and iScript advanced reverse transcriptase. Diluted cDNA, corresponding to about 10 ng of total RNA, was used per each real-time PCR (RT-PCR) reaction. According to the manufacturer’s instructions, the PCR-array gene expression profiling was performed using the gene panel Prime PCR Apoptosis (SAB Target List) H96 Assay (Bio-Rad). These predesigned arrays allow the detection of 82 genes, along with controls that include the DNA contamination control, positive PCR control, RNA quality control, and reverse transcription control. A list of the analyzed genes is reported in Figure A1. The expression levels were quantified by SYBR Green chemistry with the GeneAmp PCR System 9700 (Applied Biosystems, Foster City, CA, USA). The Ct values were exported and analyzed using Prime PCR Analysis software version 1.0 (Bio-Rad). Relative quantitation was performed using the 2^−∆∆Ct^ method, employing a panel of three housekeeping genes: glyceraldehyde-3-phosphate dehydrogenase (GAPDH), hypoxanthine phosphoribosyltransferase 1 (HPRT1), and TATA-box-binding protein (TBP). Parental NB4 clones were used as calibrator samples versus the corresponding ATO-resistant clones; untreated clones were used as calibrator samples versus clones exposed to 1 μM ATO for 48 h.

For the RT-PCR validation data, the RNA samples from the experiments were retrotranscribed using random hexamer primers and amplified with reagents from Life Technologies (Thermo Fisher Scientific). The iTaq Universal SYBR Green Supermix (Bio-Rad) was used for the RT-PCR. The sequences of the primers (validated by Sigma-Aldrich) are reported in Table A1. The reaction parameters were as follows: pre-incubation 30 s at 95 °C, followed by denaturation 15 s at 95 °C, annealing 30 s at 58 °C, extension 15 s at 72 °C, for 40 cycles. Data were normalized against the GAPDH housekeeping gene and calibrated against RNA from NB4 cells.

A quantitative TaqMan real-time PCR assay (qRT-PCR) was designed for the BCL2 expression analysis in the APL patient samples. The primer and probe sequences (designed using the Software Primer Express, Thermo Fisher Scientific) are shown in Table A2. Briefly, the RT-PCR products were cloned into the plasmid vector pCR II-TOPO (Invitrogen) to generate plasmid standard curves. After selection, the plasmid DNA was purified from recombinant colonies by the NucleoSpin Plasmid DNA purification kit (Macherey-Nagel, Milan, Italy) and sequenced using an ABI 3130 automated sequencer (Thermo Fisher Scientific). The absorbance measurement determined the plasmid DNA concentration and serial plasmid dilutions were prepared for the qRT-PCR assays to generate a standard curve. The qRT-PCR reactions and fluorescence measurements were conducted on the ABI PRISM 7700 Sequence Detection System (Thermo Fisher Scientific). The reaction parameters were as follows: pre-incubation 2 min at 50 °C plus 10 min at 95 °C, followed by 40 cycles at 95 °C for 15 s and at 60 °C for 1 min. The level of each transcript was normalized based on the number of Abelson (ABL1) and expressed as the copy number of every 10^4^ copies of ABL1 [60].

### 4.4. Statistical Analysis

Statistical analysis was performed using the GraphPad Prism 5 software and the data were reported as the mean ± SD. Statistical analysis of the IC_50_ differences between two groups were performed using the unpaired Student’s *t*-test. All the statistical tests were two-sided. Differences were considered statistically significant when *p* < 0.05. The correlations between drugs’ IC_50_ values and gene expression were examined using the non-parametric Spearman’s rank test.

## Data Availability

Data are contained within the article.

## Appendix A

**Table A1:** Primer sequences used for the relative SYBR Green RNA analysis by RT-PCR assay.

	FW	RV
**APAF1**	AAGCTCTCCAAATTGAAAGG	CCTTCTAAAAGGGAATGATCTC
**BCL2**	GATTGTGGCCTTCTTTGAG	GTTCCACAAAGGCATCC
**BCL2A1**	CAAGAAACTTCTACGACAGC	AAGCCATTTTCCTCTTCTTG
**BIRC2**	CCAACAGAAGATGTTTCAGG	ATTATACCCCTGCAAATAGGG
**BIRC3**	ACAAGCAAGAGAACTGATTG	GATCTGAAACATCTTCTGTGG
**CD70**	AATCACACAGGACCTCAG	GTCACCTGGATGTGTACC
**GAPDH**	TCGGAGTCAACGGATTTG	CAACAATATCCACTTTACCAGAG
**NOL3**	GTAGACAAGGGAACATTTGAG	ATCTACTCTTATGCCTCTGG
**IL10**	GCCTTTAATAAGCTCCAAGAG	ATCTTCATTGTCATGTAGGC
**XIAP**	AGTGTCTGGTAAGAACTACTG	CCCATTCGTATAGCTTCTTG

**Table A2:** Primer and probe sequences used for the BCL2 and ABL1 qRT-PCR assay.

	BCL2	ABL1
**FW**	ACCTGCACACCTGGATCCA	TGGAGATAACACTCTAAGCATAATAAAGGT
**Probe**	ATAACGGAGGCTGGGATG	CCATTTTTGGTTTGGGCTTCACACCATT
**RV**	GGGCCGTACAGTTCCACAAA	GATGTAGTTGCTTGGGACCCA
